# Recent Advances in Polymeric Nanoparticle-Encapsulated Drugs against Intracellular Infections

**DOI:** 10.3390/molecules25163760

**Published:** 2020-08-18

**Authors:** Arturo Sánchez, Susana P. Mejía, Jahir Orozco

**Affiliations:** 1 Max Planck Tandem Group in Nanobioengineering, University of Antioquia, Complejo Ruta N, Calle 67 Nº 52-20, Medellín 050010, Colombia; arturo.sanchez@udea.edu.co (A.S.); susanap21@gmail.com (S.P.M.); 2 Experimental and Medical Micology Group, Corporación para Investigaciones Biológicas (CIB), Carrera, 72A Nº 78B–141 Medellín 050010, Colombia

**Keywords:** polymeric nanocarriers, intracellular infections, drugs, polymeric properties, administration route, pharmacokinetics, biodistribution, nanotoxicology

## Abstract

Polymeric nanocarriers (PNs) have demonstrated to be a promising alternative to treat intracellular infections. They have outstanding performance in delivering antimicrobials intracellularly to reach an adequate dose level and improve their therapeutic efficacy. PNs offer opportunities for preventing unwanted drug interactions and degradation before reaching the target cell of tissue and thus decreasing the development of resistance in microorganisms. The use of PNs has the potential to reduce the dose and adverse side effects, providing better efficiency and effectiveness of therapeutic regimens, especially in drugs having high toxicity, low solubility in the physiological environment and low bioavailability. This review provides an overview of nanoparticles made of different polymeric precursors and the main methodologies to nanofabricate platforms of tuned physicochemical and morphological properties and surface chemistry for controlled release of antimicrobials in the target. It highlights the versatility of these nanosystems and their challenges and opportunities to deliver antimicrobial drugs to treat intracellular infections and mentions nanotoxicology aspects and future outlooks.

## 1. Introduction

In the frontier of different areas, nanochemistry uses a variety of methods to assemble materials at a nanometer-scale size, with new unique features in respect to the bulk material counterparts, including electronic, magnetic, optical, chemical, and mechanical properties. The interdisciplinarity of nanochemistry involves scientific and technical knowledge from diverse fields such as natural- and material-sciences, engineering, medicine, and pharmacy, among others. Although it is not an easy task, it is aimed at searching for new or existing (bio)materials, understanding their interactions and providing new functionalities towards new products and unexpected applications; these perspectives are encouraging and highly promising, up to the point of being able to generate a revolutionary knowledge of the world at the nanoscale. Within the health care field, nanochemistry offers outstanding opportunities for the design of diagnostic and therapeutic tools; for example, nanoconjugates and nanoplatforms assembled for the controlled and site-specific delivery of active principles with enhanced pharmacological properties against intracellular microorganisms. 

Intracellular infectious diseases represent a major challenge in health care due to the low specificity of available treatments and the appearance of co-infections and drug-resistant pathogens, which limits the existing therapies [1,2,3,4,5,6]. Recent advances in the field of nanotechnology offer alternatives to improve the biological activity of existing drugs, which is of great potential to help to overcome the drawbacks inherent in the treatment of intracellular infectious diseases. Encapsulation of drugs into nanoparticles (NP) represents a valuable option to improve drug solubility and biodistribution, prevent undesirable interactions and drug degradation before reaching the target tissues and cells [1] and non-specific accumulation of drugs in other tissues. In this context, nanoencapsulated drugs hold the potential to increase the efficiency and effectiveness of therapeutic regimens, particularly in drugs with low solubility, short half-life, variable absorption, and undesirable interactions [4,7,8,9,10,11,12]. Such improvement comes from the reduction of dose and drug frequencies in patients, which dramatically impact on decreasing both toxic and side effects that drugs usually have intrinsically. 

In the last years, the encapsulation of drugs into synthetic and natural polymeric nanocarriers (PNs) has been one of the most explored systems for drug delivery. The resultant nanosystems enjoy high biocompatibility and biodegradability, being very reproducible and amenable for mass production. PNs are usually designed to be stable, have high drug loading capacity and the ability to transport one or more active ingredients (with similar or different physicochemical properties), or a combination of therapeutic agents in the same formulation. Such features are very appealing for delivering drugs by different routes and multipurpose clinical approaches [4,13,14,15]. Size, surface charge and morphology of PNs can be tailored on demand to produce solid capsules/nanoparticles, amphiphilic structures (micelles), and hyperbranched structures (dendrimers), and vesicles [16,17,18,19,20,21,22], which sizes can usually be smaller than 100 nm but eventually reach hundreds of nm, depending upon the active ingredient or drug, the type and length of the polymer and the preparation method [23,24,25]. Besides, the polymers can be chemically modified to encapsulate, adsorb, disperse, or bound the drug [14,19,21,26,27,28]. 

Along with modulation of size, form and physicochemical properties of the polymer, to functionalize PNs with specific ligands may actively drive drugs to site-specific for the efficient drug uptaking, thereby readily reaching therapeutic intracellular levels [5,9,12,29,30,31]. Moreover, the PNs are extremely versatile in controlling the drug release profile. For instance, PNs can be tuned for the immediate or sustained delivery of drugs in a localized place either by natural diffusion of the drug or by osmotic, erosion or degradation processes. However, such mechanisms have evolved towards more refined ones based on physical, chemical, or biochemical external stimuli. In this context, triggered local changes in pH and temperature, in the number of reactive oxygen species (ROS), the reductive or oxidative states, and conformational changes in the polymer have been extensively used [14,15,32].

In recent years, a large number of drug delivery systems have been described in many medical areas, including intracellular infections [3,5,7,8,10,18,29,30,33]. Indeed, the global infectious disease therapeutics market size was valued at $46.88 billion in 2018 and is estimated to reach $64.5 in 2023 [34]. Yet, many challenges are still needed to face before many antimicrobial-based nanoformulations hit the market. They are related to the fact that pathogenic microorganisms are established mainly in phagocytic cells. Besides, nanocarriers injected intravenously are mostly recognized and cleaned either by phagocytic cells from the endothelial reticulum system (RES) or by the mononuclear phagocyte system (MPS) going “passively” to the macrophages, the reservoir of most intracellular pathogens [35]. Another challenge of nanocarriers is the necessity to increase their drug loading capacity to administrate less amount of material but sufficient to reach a therapeutic concentration of drug at the site of infection that avoids toxicity and side effects [33,36,37,38,39]. Other imminent necessities are related to the increase of stability and better control of the drug release profile. Therefore, it is of paramount importance to design antimicrobial-based nanocarriers, not only with high drug loading capacity and well-controlled release but site-directed to infected cells.

This review provides an overview of the use of different organic nanoparticle precursors and the main assembly methods to produce nanoplatforms of tuned physicochemical and morphological properties and surface chemistry for controlled release of antimicrobials in a target cell or tissue, in a physiological environment, depending on the active principle nature and administration route. It points out the remarkable versatility of these nanosystems and details the facing challenges, as well as the opportunities of the nanoplatforms to deliver antimicrobial drugs to efficiently and effectively treat intracellular infections. It finally mentions some nanotoxicology aspects essential to be considered and prospects in the topic that expect to stimulate advances and new opportunities in this promising field. 

## 2. Polymeric Nanocarriers against Intracellular Infections: General Aspects

The efficient treatment of intracellular infections by antimicrobials is highly challenging. Challenges are related to the evasion of intracellular infectious agents by host phagocytic killing mechanisms, the establishment of intracellular survival machinery and the worldwide misuse of antibiotics, which are rising multidrug resistance of pathogens [40,41]. Besides, many conventional antimicrobial-based treatments possess low cellular penetration and therefore, drug distribution at the subcellular level is not uniform; thus, the site of infection may remain without treatment. Once inside the cell, antimicrobials activity can be influenced by enzymatic inactivation, and changes in pH and chemical environment, as discussed in the next sections. It may result in a low intracellular concentration of antimicrobials, thus limiting the efficacy and efficiency of the therapy [42,43].

PNs are an alternative approach extensively studied to overcome some limitations of antimicrobial therapies by encapsulating drugs to improve their ability to enter the cells and release the cargo intracellularly [44,45,46]. As detailed in the following sections, cellular internalization of PNs includes phagocytotic-mediated-, clathrin-mediated-, caveolae-mediated- and receptor-mediated-endocytosis or a mixture of them [45,47,48]. After cellular uptake, it engineers the escape of endocytic vesicles formed to avoid lysosomal degradation for cytosolic delivery as well as the possible intracellular trafficking of PNs targeting subcellular compartments where determined intracellular pathogens reside. PNs protect antimicrobials of degradation, increase their solubility and bioavailability for their controlled and targeted release. 

They may reduce drug dose and adverse side effects and provide better efficiency and efficacy of therapeutic regimens. By modulating properties and functionality of PNs they can specifically address the target tissue or cell depending on the route of administration (oral, parenteral, intranasal, topical, and intravenous, among others).

PNs are commonly built by self-assembly of biocompatible and biodegradable polymeric materials to minimize non-specific cytotoxicity on healthy tissues with convenient degradation kinetic profiles and complete metabolization of degradation products [15,49,50]. A myriad of nanoplatforms with specific morphological characteristics (nanorods, nanoworms, nanodiscs, among others), size and surface chemistry, can be produced depending on a diversity of precursors, fabrication methods and antimicrobials’ chemical nature. Among them, nanocapsules and nanospheres are the most common. While nanocapsules of vesicular structure generally have the drug immersed in a liquid core surrounded by a solidified polymeric shell, nanospheres are a solid/mass polymeric matrix in which drug is encapsulated inside or over the structure surface. Active principles can be associated with nanostructures by physical encapsulation, covalent conjugation, adsorption, electrostatic- and van der Waal-interactions. Modulation of cellular uptake, extracellular transport and intracellular drug delivery is achieved by tuning size, shape, surface chemistry, antimicrobials’ intrinsic properties and microenvironments that PNs need to overstep. Targeting can be passive, activated by ligands attached to the outermost nanoparticle surface and drug release by natural ways or triggered by an external or internal stimulus [45,51,52]. 

In nanotherapeutics based on passive targeting, nanocarriers must reach the site of action by physiological or physicochemical changes that occur naturally in the body. They include differences in the pH among tissues (tumor microenvironment), defective vasculature enhanced permeability and retention effect (EPR), capture by the mononuclear phagocytic system and differences in the redox properties of the systems. In active targeting, the surface of nanocarriers is modified to generate affinity with bioreceptors or cellular biomarkers, tissues or organs through ligand-receptor interactions [46,53]. Ligand-nanocarrier conjugation uses different coupling methodologies, including the formation of disulfide bonds, cross-linking, covalent coupling, ionic interactions, layer-by-layer assembly, among other strategies [54]. Coating the nanocarriers with targeting ligands such as peptides, antibodies, lectins, sugars, folate- and mannose-receptors, among others, drive them to the site of action, thereby increasing the concentration of the active principle in the place and avoiding non-specific accumulations and the concomitant adverse effects [55,56,57].

Drugs are commonly released from nanocapsules rapidly or in a sustained fashion. It depends on the formulation. Drug release can be by diffusion through the porous or polymeric chains of the PNs or by osmotic-, erosion- or degradation-processes. However, natural release mechanisms are migrating towards more sophisticated ones based on physical, chemical or biochemical external stimuli. For example, the response of PNs can be triggered by stimulation with radiation, ultrasound, magnetic fields and temperature. The response can be modulated by changes in the pH and ionic strength, the cellular environment, or by enzymes [58]. Among stimuli-responsive PNs, nanogel-based PNs establish three-dimensional polymeric networks at nanoscale level with high capacity to water uptake and cross-linking. While they change their volume by absorbing water, the surrounding environment influences their behavior, thus generating stimuli-responsive systems (pH, ionic strength, electric field or temperature). Nanogels are prepared by natural or synthetic polymers, including chitosan, methylcellulose, ethylcellulose, dextran, polysaccharide-based polymers, dextrin, poly(oleic acid-Y-N-isopropyl acrylamide), polyvinyl alcohol, alginate, hyaluronic acid, poly(N-isopropyl acrylamide), among others. Nanoencapsulation of active principles of different natures can be achieved either by nanohydrogels or by nano-organo-gels, having higher loading capacity, controlled release and biocompatibility regarding other types of nanoparticles.

Nanohydrogels encapsulate hydrophobic and hydrophilic compounds, where the cross-linked network functions as a matrix holding the absorbed liquid medium, which modulates the diffusion of the active principles [59]. In contrast, nano-organo-gels have a micelle-like structure having hydrophobic regions (that hold oily compounds) attached to the hydrophilic regions at the center of the nanostructure. Nanogels performance can be modulated by changing their size and surface charge, or by incorporating targeting ligands, changes in cross-linking density or PEGylation strategies. Encapsulated active principles are released by hydrolytic degradation of the gel network. Recent studies have demonstrated the disruption of nanogels by using cross-linking agents sensible to temperature, light, differences in pH, use of disulfide bond linkages and cleavage by glutathione (GSH) enzyme. Therefore, the active principle can reach intracellular targets after nanogels are endocytosed, promoting endolysosome escape and improving intracellular therapy [59,60,61].

### 2.1. Chemical Nature of the Polymer, Physicochemical Properties and Interactions

**Figure 1:**
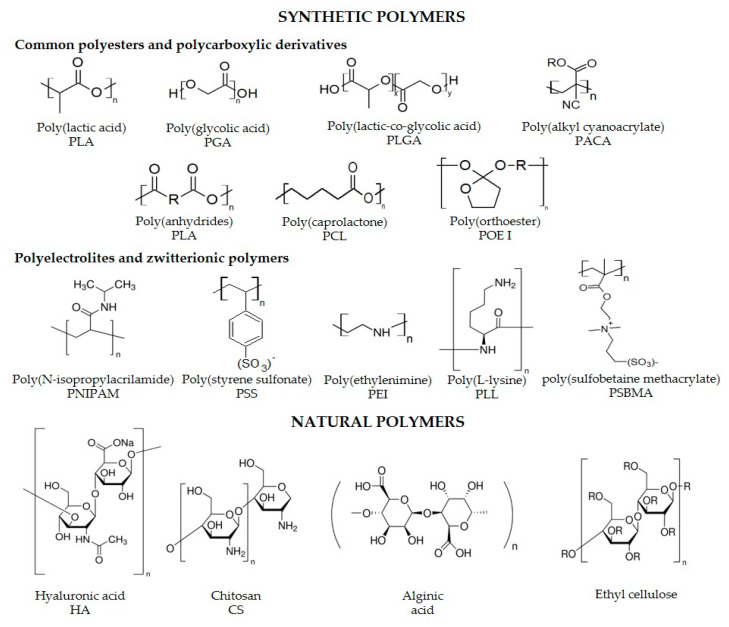
Polymers commonly used to manufacture PNs (polymeric nanocarriers).

PNs can be obtained from natural or synthetic polymers, having specific physicochemical features derived from the polymer chemical properties and interactions when assembling the nanostructures. Numerous types of biocompatible and biodegradable polymers from natural or synthetic sources have been reviewed elsewhere [62,63,64,65]. Among natural polymers, some typical examples include chitosan (CS), dextran, alginate, hyaluronic acid, natural polyelectrolytes such as protamine sulfate (PS), alginic acid, cellulose sulfate, dextran sulfate, heparin and carboxymethylcellulose. These polymers have excellent biocompatible/biodegradable properties because they come from natural sources and their degradation products are easily metabolizable [62,66,67,68,69]. Common degradable and biodegradable polymers of synthetic sources are summarized in Figure 1. Among biocompatible polymers, poly(lactic-co-glycolic acid) PLGA has been one of the most extended; comments on PLGA properties as a model of biocompatible polymer are as follows.

PLGA has excellent biodegradability, biocompatibility, mechanical strength, stability under physiological conditions, low toxicity, and amenability for controlled release. Indeed, there are currently pharmaceutical products based on this polymer, which already have a long history of clinical use [15]. PLGA degradation products in the physiological environment are lactic and glycolic acid, which can be metabolized through the Krebs cycle in carbon dioxide and water. Figure 1 shows the PLGA chemical structure, where x is the number of lactic acid units and y is the number of glycolic acid units. PLGA nanocapsules of different sizes and morphologies have been used for encapsulation of different types of hydrophilic and hydrophobic therapeutic molecules (DNA, peptides, proteins, antibiotics, antifungals, among others) with a wide range of active principle molecular weight [70,71].

PLGA is synthesized by ring-opening polymerization from different ratios of the lactic and glycolic acid monomers, using 2-ethyl hexanoate tin (II) and tin (II) alkoxides as catalysts, thus forming the characteristic PLGA ester linkages. Typical examples are PLGA 75:25, PLGA 65:25 and PLGA 50:50, indicating the lactic: glycolic acid ratio, respectively [71]. The versatility of PLGA is indeed coming from the different intrinsic properties of the polymers, which composition is impacting crystallinity. For example, PLA is chiral, so it exists in various forms such as PLLA (poly-l-lactic acid), PDLA (poly-d-lactic acid) and PDLLA (poly-d-l-lactic acid). Whereas PLLA has been reported to be highly crystalline, PDLA is entirely amorphous, and PDLLA is amorphous. In contrast, PGA is not chiral and highly crystalline. When copolymers of PLGA are prepared from PLLA and PGA, the resultant polymer has a crystalline character, but whether copolymers are made from PGA and PDLA, the amorphous character of the latter predominates producing an amorphous polymer. If PDLLA and PGA are used, the polymer obtained is amorphous [15,57,71].

Crystallinity properties have a direct effect on biodegradation, e.g., amorphous polymers biodegrade better in a cellular environment with less energy, as compared to polymers with higher crystalline arrangement needing higher energy. This fact explains the extended use of poly-d,l-lactic-co-glycolic acid, hereafter called PLGA, of improved amorphous character regarding PDLLA, for biomedical applications. It is worth mentioning that lactic acid is more hydrophobic than glycolic acid; therefore, polymers having a higher lactic:glycolic acid ratio have higher hydrophobicity, absorb less water and suffer slower degradation in a physiological environment. Besides, PLGA copolymer’s glass transition temperatures (Tg) are above 37 °C, so they are stable enough for administration in a physiological environment. Decreasing the copolymer molecular weight and lactic acid composition causes a decrease in glass transition temperature and, therefore, in polymer stiffness. Molecular weight also has a direct effect on particle size and other properties. For instance, the lower the molecular weight, the smaller the particle size, the higher the degradation rate, the higher the maximum concentration in plasma and, therefore, the less accumulation in organs. Furthermore, the higher the PGA composition in PLGA, the greater the hydrophilicity and, therefore, the greater the degradation rate. Overall, physical properties such as size, composition, molecular weight, glass transition temperature and degree of crystallinity affect polymer mechanical strength and influence its stability and biodegradation profile [15]. 

Applications of these polymers are based on exploiting specific properties, e.g., both cellular uptake and cytosolic delivery of drugs are relevant to treat intracellular infections. In this context, poly(alkyl acrylic acid) and poly(alkyl acrylic acid-co-alkyl acrylate) are amphiphilic synthetic pH-responsive polymers that can disrupt membranes. Carboxyl groups and hydrophobic alkyl groups from the polymers may be protonated in endosomes. Then, hydrophobicity of this type of polymers increases when pH decreases, facilitating the penetration of the polymer into the endosomal membrane, disrupting it, thus improving the intracellular delivery of drugs [72,73,74]. 

Biocompatibility, biodegradability and safety of the polymer are essential features to consider when designing PNs. In this sense, the natural polymer CS and its derivatives deserve to be commented as relevant platforms for nanotherapeutic applications, including antimicrobial therapies [75,76,77]. CS is classified by the FDA as “generally regarded as safe.” It has been extensively studied in nanotherapeutics and nowadays, there are marketed CS-based formulations in nutraceutical and medical devices [44,78]. CS is a cationic linear polysaccharide copolymer made of glucosamine and N-acetylglucosamine. The number of acetylated monomers and how they are distributed in the polymeric chains profoundly impact the solubility of CS and its conformation in aqueous solutions. CS is mostly hydrophilic, has a pH-dependent behavior in solution and CS-based PNs are versatile regarding nanofabrication methods. CS has mucoadhesive properties, is capable of opening tight epithelial junctions, and can be chemically functionalized in its amino or hydroxyl groups [75,79,80,81]. Properties of CS polymers define the final fate of the resultant nanoparticulate system. Then, molecular weight, crystallinity, chemical nature, as well as terminal end group, viscosity, deacetylation degree and electrical charge of CS are worthy of consideration. In the case of prodrugs, drugs covalently conjugated with polymers; engineering suitable linkers, enzyme-, pH- or temperature-triggering PN-based systems is crucial based on the cargo-release mechanisms. In this context, reduction-sensitive linkers having disulfide or thioether bonding, acid-sensitive linkers having hydrazone and acetal moieties, thermally responsive polymers, such as poly(N-isopropyl acrylamide) are available to build stimuli-responsive prodrug-based PNs. Besides, interactions among nanoformulation components are significant to consider when designing PNs to deliver in vitro and in vivo drugs intracellularly.

### 2.2. Colloidal Properties of PNs for Intracellular Therapy

When establishing PN-cell interface interactions, the driven forces involved in cell uptaking are inherent to PNs (size, shape, elasticity and surface chemistry) and cell membrane characteristics (elasticity, bioreceptors), etc. In general, cellular uptake results are selective to size, sensitive to shape and stiffness-dependent [48,64,82,83,84,85]. For instance, nanocarriers need a long circulation time in plasma, evading MPS and being further in contact with the target tissue, which can be modulated by a rational choice of nanoparticle size. Whereas MPS recognizes and removes particles larger than 10 nm, the kidney removes small particles (<10 nm) and phagocytic uptake takes place in particles of larger size (i.e., nanoparticles ranging 200–1500 nm) [48,85,86]. Nanocarriers’ size and size particle distribution impact the time of circulation in plasma, and further penetration and accumulation in tissues, as well as biodistribution, to finally drug release at the intracellular level. Particles of 100 nm can avoid MPS more efficiently, thus increasing the circulation time in blood as compared to bigger particles [84,85]. The final fate of nanoparticles is also affected by the type of intracellular pathogen, cellular microenvironments where the intracellular pathogens are found, and stage of the disease. Reports have demonstrated that “protein corona” is NPs’ size-dependent [87] and that penetration of nanocarriers and accumulation in tumors is promoted by nanocarriers having smaller sizes in a range of 50–200 nm by the EPR effect. For more detailed information, we will come back to this point in the following sections.

Particle size distribution indicated by the polydispersity index (PDI) determines how monodisperse is the system and, therefore, how similar are the particle size to each other. Uneven particle size can cause variable pharmacokinetic parameters affecting formulation therapeutic efficiency [88]. In general, PDI ≤ 0.1 are highly monodisperse; between 0.1 and 0.4 are moderately polydisperse and greater than 0.4 are highly polydisperse [89]. Therefore, particle size and distribution and nanoparticle clearance mechanisms are crucial when designing a formulation [84,85]. For instance, Toti et al. reported nanoparticles formulated with PLGA ranging from 100 to 300 nm that were efficiently internalized into mammalian endothelial cells through pinocytosis and endocytosis for sustained drug release. In this study, rifampin and azithromycin were encapsulated in PLGA as a treatment strategy against intracellular bacterial pathogens *Chlamydia trachomatis* and *Chlamydia Pneumoniae*. Nanoparticles with an average size of 260 nm had sustained release of drugs intracellularly, demonstrating its effectiveness [90]. 

Nanoparticles with different shapes, geometry and aspect ratio have different behavior when they are flowing in the plasma. For instance, discoidal particles favor vessel wall interactions higher than spherical counterparts due to their tumbling and margination dynamics with higher adhesion to endothelium and particle binding [84]. Nanodiscs, nanorods, and nanoworms, showed reduced phagocytosis, different biodistribution behavior, and prolonged plasma circulation as compared to spherical nanoparticles. Besides, discoidal nanoparticles accumulated mostly in highly vascularized organs such as lungs and spleen. Prolate ellipsoid particles showed higher coupling to macrophages than oblate ellipsoid or spherical particles. Adhesion and margination processes are significatively affected by the shape, because of the higher surface area of asymmetric nanoparticles as compared to spherical ones. Therefore, nanoparticles with discoidal and rod shapes can be designed for targeting vascular endothelium with improved interaction [84,85]. 

**Figure 2:**
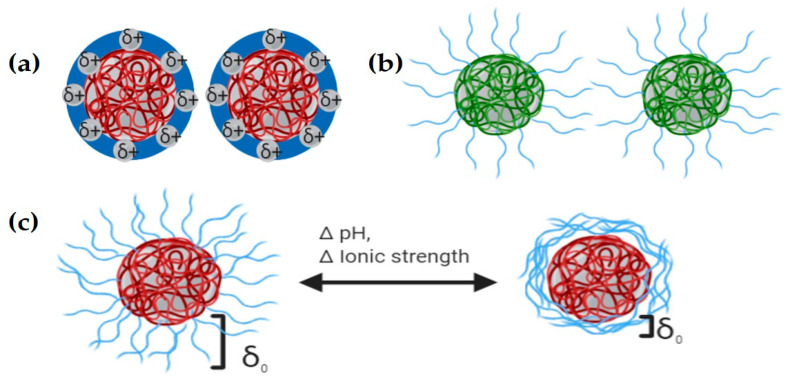
Mechanisms for colloidal stabilization of nanoparticles. (**a**) Electrostatic stabilization, δ_+_ is the electric charge generating electrostatic repulsive forces among particles. (**b**) Steric stabilization. (**c**) Electrosteric stabilization as a function of pH and ionic strength, δ_0_ is the thickness of polyelectrolyte.

A key point in nanotherapeutics development is the stabilization of nanocarriers to preserve their physicochemical properties and performance characteristics. Previous studies reported that the colloidal stability of suspended PNs is defined by electrostatic, steric, or electrosteric effects among particles to overcome van der Waals attractive forces (Figure 2). Nanoparticle’ surface charge depends not only on the particle surface properties but on the solution conditions such as ionic strength and pH and can be estimated by Electrophoretic Light Scattering (ELS) [89,91]. At high absolute values of ζ potential (≥30 mV), there is an electrostatic repulsion stabilization preventing nanoparticle agglomeration (Figure 2a). A surfactant or other organic species adsorption produces a layer with enough thickness and density to prevent agglomeration due to steric hindrance (Figure 2b). Polyelectrolytes adsorption containing ionizable groups over the surface of the particles can stabilize them in an electrosteric manner (electrostatic and steric), preventing agglomeration by both mechanisms. Therefore, conditions such as pH, ionic strength, molecular weight and concentration are the most influencing parameters on nanoparticle stability (Figure 2c) [91]. Nanoparticles’ surface charge takes part in interaction with the cell membrane (negatively charged) and, therefore, in the uptake mechanism. This interaction is the most energetically favorable regarding, for example, hydrophobic/hydrophilic nanoparticle/cell interactions. Positively charged nanoparticles are highly and unspecifically uptaken by a variety of cells and once internalized, they can escape from the endosome and release their cargo [84]. Macrophages more easily and selectively internalize negatively charged nanoparticles than nanoparticles with neutral surfaces [92], but both types of nanoparticles (negatively charged and neutral) remain in the lysosome [93]. Switchable surface charge nanoparticles made of zwitterionic polymers with pH-sensitive pendant groups can be uptaken when they are around the cellular microenvironment with acidic pH, transforming its negative surface charge to cationic surface charge promoting the endocytosis mechanism [84,94]. Surface charge is involved in the opsonization process and the type of plasma proteins adsorbed on top of PNs; besides, neutral nanoparticles are involved in more extended plasma circulation. Cationic and anionic particles are taken up by Kupfer cells showing higher accumulation in the liver resulting in different patterns of biodistribution. Intracellular trafficking is affected by surface charge and surface ligands, converting this feature in an effective design strategy in the case of the endosomal escape to promote cytosolic delivery of therapeutic cargo or to target a specific organelle where an intracellular pathogen can be located [48]. For instance, nanoparticles with a high buffering capacity build of polymers such as polyethyleneimine (PEI) and histidine through the “proton sponge” mechanism causes an influx of water and swelling that finally disrupts the endosome [84,85].

### 2.3. Nanoparticle Fabrication Methods

**Figure 3:**
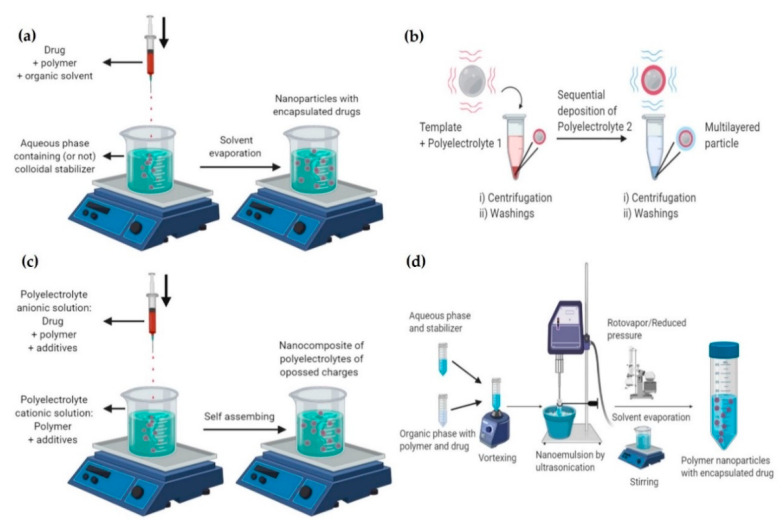
Manufacturing PNs by conventional methods. (**a**) Nanoprecipitation. The polymer dissolved in the organic solvent is added to an aqueous solution by dripping under constant stirring, forming the PNs instantly. (**b**) Layer-by-layer assembly. The solid form of the active principle is used as the core and the respective layers are formed on top, one by one, according to the electric charge. (**c**) Ionic gelation method. Self-assembling of polyelectrolytes by electrostatic interactions forming polyelectrolytic complexes. (**d**) Emulsion evaporation method. After nanoemulsion formation, the solvent evaporates gradually under reduced pressure or stirring to produce polymeric nanoparticles.

Nanoencapsulation of therapeutic principles is carried out by nanofabrication processes classified as “top-down”, “bottom-up,” or hybrids. These strategies have made it possible to obtain more efficient therapeutic formulations through highly precise control of composition, size, morphology and surface functionality [52]. Bottom-up techniques focus on the material assembly from its atoms and molecules, generally using chemical procedures, until the formation of a nano-sized conglomerate. “Top-down” techniques consist of designing and miniaturizing structure size to obtain functional systems at the nanoscale, involving the use of microfabrication technologies such as electron beam lithography and nanoimprint lithography, among others, which allow nanostructure molding at the atomic level. PNs are commonly nanostructured at laboratory scale by bottom-up techniques in which diblock copolymers containing segments of hydrophobic and hydrophilic nature are self-assembled. The most extended methods for nanoparticle fabrication are nanoprecipitation, nanoemulsion, LbL assembly, ionic gelation, and polymerization. Only some relevant bottom-up methods are discussed herein, as these methodologies have been extensively reviewed elsewhere (Figure 3) [15,51,52,62,95].

#### 2.3.1. Nanoprecipitation

Preparation of PNs by nanoprecipitation (Figure 3a) requires the mixing of two phases (solvent and non-solvent) with the precursors (drug, polymer, stabilizer) dissolved in either of them or both, by taking advantage of gelling and solubility properties of the polymers. The self-assembly of a hydrophobic polymer in a non-solvent phase allows to separate phases and, therefore, the nanoprecipitation. In this case, the organic phase is prepared to dissolve polymer and drug in an organic solvent, volatile or not, or in a mixture of solvents. After this, it is added to an aqueous phase (non-solvent) with or without a colloidal stabilizer. The addition of the organic phase to the aqueous phase in a volume-controlled manner (usually by using a syringe) produce drops with smaller and controlled size. This procedure leads to the co-precipitation of the nanoparticles when the solvent is removed by evaporation or by agitation, whether it is volatile or by dialysis if it is not volatile. Extensive research involving encapsulating hydrophobic or hydrophilic active principles into polymeric nanoparticles by nanoprecipitation to treat intracellular infections have been conducted [51,62]. Common polymers included synthetic ones (PLA, PLGA, PACA), PEGylated ones (PLA-PEG, PLGA-PEG), PEI, and natural polymers (CS, alginate, hyaluronan-poly (g-benzyl-l-glutamate), and more recently squalenic acid. The nanoprecipitation mechanism depends on the polymer and the drug associated in the solvent/water mixture to form small aggregates at supersaturation critical concentration. Once critical supersaturation is reached, nuclei are formed by self-assembly and the growth of particles is then limited by diffusion of molecules to the nucleus surface. The process is complex and previous reports have demonstrated the influence of several parameters on the colloidal characteristics of the formulation such as the solvent/non-solvent mixing time, molecular weight of the polymer, type of solvent, solvent/water ratio, drug/polymer ratio, and interfacial tension as reviewed elsewhere [51,96].

#### 2.3.2. Layer-by-Layer (LbL)

LbL technique (Figure 3b), uses the sequential adsorption of polyelectrolytes, charged molecules or nanostructures to produce polymeric nanoparticles while encapsulating active principles [97]. Whereas polyanion/polycation interaction allows the building of multilayer structures, surfactants can be added to stabilize the nanoparticles. Conventionally, the substrate or active principle to encapsulate is immersed in a polyelectrolyte solution with a compatible electrical charge to produce interactions that promotes the active principle association. Interactions include hydrogen, ionic and covalent bonding, electrostatic interactions and biologic specific interactions. Several layers of opposite charge are then formed on top of the active principle with washing steps in between. Multiple strategies based on the LbL approach, including functionalization of the external layer with ligands to be specifically recognized by the target cells, have been explored for targeting intracellular infections. The molecular weight of polyelectrolytes used in this methodology for the shell assembly is limited to be ≤65 KDa due to restricted space for adsorption of the polymeric chain when it is nanostructured [97]. Commonly synthetic and natural polyelectrolytes are summarized in Figure 1.

#### 2.3.3. Ionic Gelation

Coacervation or ionic gelation method is founded on electrostatic interactions as it is the case of CS nanoparticle systems in which the cationic groups formed by positively charged amino groups interact with anionic groups belonging to tripolyphosphates forming coacervates [95,98,99] (Figure 3c). The process can be classified as simple or complex coacervation depending on the number of macromolecules employed. The separation of a macromolecular solution to form the dense coacervate basically comprises the following steps in constant agitation. In the first step, it is necessary to disperse the active principle into a solution of a surface-active hydrocolloid to obtain precipitation of the hydrocolloid further. The precipitation can be done by changing the pH, a non-solvent, the temperature or the use of electrolytes. The addition of other hydrocolloids to the solution makes possible the formation of a polymer-polymer complex in the case of complex coacervation. The final step consists of hardening or stabilization by cross-linking agents (i.e., glutaraldehyde, transglutaminase) to obtain the polymeric nanoparticles. Parameters influencing the colloidal properties of the resultant PNs (size and particle distribution) include the molecular weight, viscosity of the non-solvent, amount of cross-linking agent, among others. This method has been broadly used for nanoencapsulation of hydrophilic molecules and proteins as reviewed elsewhere [62,75,80,98,99,100].

#### 2.3.4. Emulsification-Evaporation

Hydrophobic compounds are commonly encapsulated into polymeric nanocarriers by the Emulsification-Evaporation Method (EEM) using single oil-in-water (O/W) emulsion (Figure 3d) [101]. EEM method consists of obtaining an O/W nanoemulsion and then removal of solvent to obtain nanocarriers in suspension. The organic phase is composed of a water-immiscible solvent, the active principle to be encapsulated and a preformed polymer, and the aqueous phase contains a surfactant or stabilizing agent. Whereas high shear force agitation produces O/W nanoemulsion, high or low energy can be used to size-control and then for obtaining nanoemulsion or microemulsions. Removal of the organic solvent causes polymer aggregation, forming the particles.

The first steps in nanoemulsion preparation using high energy methods are macro droplet formation, deformation and interruption of macro droplets towards nanometric-size ones and surfactant adsorption at the interface to stabilize them by steric effect [102,103]. After solvent removal from the system, the polymer precipitates towards the water-solvent interface, while the stabilizing agents prevent flocculation and coagulation of the polymeric nanoparticles by steric and/or electrostatic repulsion. Nanoparticles are purified to remove the remaining polymer, stabilizer, residual solvent, and not encapsulated active principle by dialysis, centrifugation, tangential filtration, among others [104,105,106].

Recent advances in nanofabrication methods include 3D nanofabrication technology, where the objects are built additively by using cross-sectional layer methods. Besides, updated microfluidic systems such as flash nanoprecipitation (FNP) offer precise control at the nanoscale with multiple applications in the development of PN-based therapeutic systems [52,107,108,109]. Microfluidics require only very small volumes of nanoparticle precursors and provides precise control of phases mixing to produce reproducible PNs with different physicochemical properties [62]. In this context, antifungal itraconazole nanoencapsulated by FNP with different amphiphilic stabilizers has been studied and their mobility on nanoparticles’ surface and physical storage stability evaluated [110]. Although still in its infancy, other novel approaches involve electrohydrodynamic atomization-based methodologies, where liquid droplets are generated by the application of a large electrical potential difference. Based on this approach, PCL NPs have been produced and evaluation of critical parameters in the electrohydrodynamic process has been made to better understand the mechanism of NPs formation [23,109,111].

### 2.4. Nanoparticles Drug Release

PNs release therapeutic principles by transporting them from some initial position in the polymer matrix to the outer surface and, subsequently, to the environment in which nanoparticles are found. Drug release follows different release patterns, or a mixture of them, explained by mathematical modeling. Mathematical models allow predicting active principle release rates and their performance to optimize the formulation towards a more precise dosage regimen that minimizes adverse effects and costs. Mathematical models are varied and classified as empirical/semi-empirical and mechanistic models. Empirical/semi-empirical models are purely mathematical descriptions and are not based on any chemical, physical or biological phenomenon; neither reveals factors controlling active principle release and its predictive power is generally low. Yet, they are useful to describe different stages of the active principle release process. In contrast, mechanistic mathematical models are based on real phenomena such as diffusion (based on Fick’s law), degradation and erosion, and are useful in understanding active principle release mechanisms.

**Figure 4:**
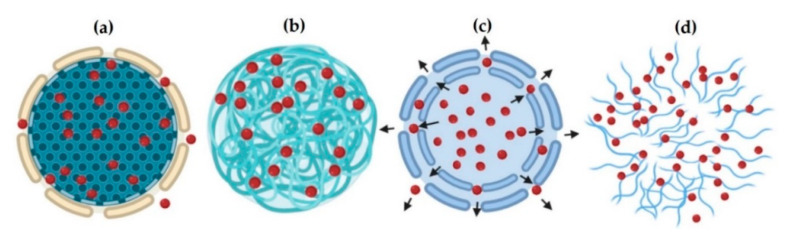
Mechanisms of release of active principles by polymeric nanocarriers. (**a**) Diffusion through water-filled pores. (**b**) Diffusion through the polymeric matrix. (**c**) Osmotic pumping. (**d**) Erosion and degradation of polymeric matrix caused by hydrolysis or for external stimulus.

It is important to note that there is a considerable variety of release mathematical models, including the contribution of Fickian and non-Fickian processes, distribution functions, such as Korsmeyer-Peppas, Higuchi, zero-order, Lindner-Lippold, Ritger-Peppas, Peppas-Sahlin, Weibull, and Monte Carlo technique, among others [112,113]. However, current models are not robust enough to describe the complexity of the active principles delivery phenomenon since there are multiple interactions and conditions established from the surrounding environment affecting the process [15,62,71,113]. Overall, drug release is governed generally by three main mechanisms: (i) Standard diffusion-controlled release; (ii) Degradation of nanoparticles from biodegradable polymers; and (iii) Release triggered by environmental conditions such as pH, temperature or radiation, in sensitive-to-stimuli PNs [62]. The process starts when the polymer rapidly absorbs water from its environment through its porous structure, occupying a volume in the polymer matrix. When the pores increase in size and number, the active principle diffuses through and the molecules move by a concentration gradient (Figure 4a). The transport can also be given by direct diffusion of the active principle through the polymeric chains, for example, in the encapsulation of small hydrophobic molecules (Figure 4b). This is the case of non-degradable reservoir-type delivery systems where the release rate is not affected by concentration gradients but by the properties of the polymeric membrane (permeability and thickness) [15]. The nanostructure porosity can produce osmotic pressure that allows active principle transportation, in a process known as osmotic pumping (Figure 4c). The diffusion-controlled release of the active principle depends on the value of its effective diffusion coefficient through the polymer matrix [114].

Stimulus-sensitive nanoparticles (Figure 4d) can change their chemical structure by employing heat, ultrasound, magnetic field, or light, degrading the nanostructure by erosion. An example of these nanostructures uses thermosensitive polymers such as poly (N-isopropyl acrylamide) and poly (vinyl-caprolactam) [15].

## 3. Challenges and Opportunities of Polymeric Nanoparticles in Treating Intracellular Pathogens

### 3.1. Intracellular Pathogens

Infectious diseases are a major health public problem, being the second cause of death worldwide [115,116,117]. Among infectious diseases, those caused by intracellular microorganisms are characterized by the ability to alter some defense functions of the host cell, affecting its combat strategies, and allowing microorganisms to survive at the intracellular level. Thanks to these strategies, all viruses (e.g., Coronavirus (COVID-19), VIH-SIDA) certain bacteria (e.g., *Mycobacterium tuberculosis, Listeria monocytogenes*, and *Salmonella typhi*), some protozoa (e.g., *Toxoplasma gondii*, *Plasmodium falsiparum* and *Leishmania* spp.), and a few fungi (such as *Histoplasma capsulatum*, *Paracoccidioides brasiliensis*) can survive inside mononuclear phagocytes (especially macrophages and dendritic cells), the more efficient immune cells to eliminate the microorganisms. Likewise, the intracellular environment protects the microorganisms against attack mechanisms of the humoral immune response, such as antibodies [29,30,35,39,118,119,120].

Some intracellular microorganisms have lost the ability to live outside their host, with whom they always have intimate and dependent relationships [35,121,122,123]. However, the disruption of the host-microorganism relationship [121] may develop a disease by escaping host response mechanisms. In contrast, facultative intracellular microorganisms have a more varied relationship with their hosts, retain the ability to replicate outside them and can colonize different cell types and diverse extracellular environments [5,30,35,121,124]. They have regulatory systems that are responsible for reprogramming their physiology during the transition from the extracellular to the intracellular environment or vice versa, whose host-microorganism interaction is usually related to the immune status of the host [121,124].

Antimicrobials are powerful drugs to fight infectious diseases, but there exist relatively few options in the market [125,126]. Indeed, according to data reported by the FDA, their production has been decreasing since 1983 [125,127]. Antimicrobials kill the microorganisms or inhibit their reproduction, which is further eliminated by the host immune system. Their intake in an adequate manner saves lives, while following the proper indications, even after the symptoms are gone, is key to curing and preventing the development of resistant microorganisms [128,129]. Depending on the type of infection, there exist specific antimicrobials to fight microorganisms efficiently. The infections caused by bacteria, fungus, viruses, or parasites are treated with antibiotics, antifungals, antivirals and antiparasitics, respectively. Each antimicrobial presents different challenges, mostly related to the biology of the microorganisms and the pathogenesis of the diseases [1,9,129,130,131]. Physicochemical properties of antimicrobials (solubility, molecular weight, hydrophobicity/hydrophilicity, charge, and alkalinity) are important parameters to predict drug-pathogens action. They define, for instance, whether they are stable under physiological conditions, which can dramatically impact the efficacy and effectiveness of the treatment. Table 1 summarizes the clinically relevant therapeutic principles to treat intracellular infections, indicating the type, target, action mechanism, adverse effects and reference.

The development of drug resistance has occurred on a large scale over time, reducing the effectiveness of treatments. Microorganisms produce resistance by acquiring the expression of resistance genes or by selecting microorganisms that express them, which gene acquisition can take place spontaneously or by horizontal transfer usually in response to drug exposure [132,133,134]. The selective pressure in favor of the microorganisms that present the resistant genes occurs, in part, due to poor adherence to treatment by patients, the use of antibiotics for long periods, or the low drug microbicidal effect. When a patient does not take the adequate drug dose, there is an increase in the selective pressure in favor of the resistance genes because the microorganisms that present them are exposed to the drug, but it cannot eliminate them [132,135]. Table 2 summarizes some examples of antimicrobial resistance.

### 3.2. Biological Barriers

Drugs used for the treatment of intracellular infection diseases are typically administered by oral, intravenous (IV) and inhalation routes. Depending on the administration route, drugs are exposed to several challenges due to the existence of biological barriers in the human body. The biological barriers exist to maintain homeostasis, which prevents any foreign substance and pathogen [87,162,163,164]. The therapeutic efficacy of the drug is affected by interaction with biological barriers, which is related to the drug’s physicochemical properties and may alter drug biodistribution and pharmacokinetics. Nanocarriers suffer from the same issue with the biological barriers; thus, modulation of physicochemical properties of the nanocarriers is necessary to facilitate surpassing biological barriers [87,162,163,164].

#### 3.2.1. Intravenous Administration

IV is an invasive administration route with the inconvenience of having high costs and produce adverse effects. The major barriers to the IV route treatment include the liver and the spleen, which together are the clearing organ, forming a dynamic barrier to keep harmful substances out of the body. The organs in their capillaries have many mononuclear phagocytes, which constitute the reticuloendothelial system (RES) [87,162,164]. The macrophages are always on the lookout for any foreign substance to get rid of. Drugs administrated by IV follow the path circulation, margination, and cell internalization. In circulation, plasma proteins (albumin, complements, immunoglobulins, and apolipoproteins) are adsorbed onto the therapeutic agents to form a corona in a process known as opsonization. The type of proteins that bind to the agents depends on the size, superficial charge, and hydrophobicity/hydrophilicity. Depending on the type of proteins adsorbed as protein corona, the therapeutic agent can be recognized by the macrophages from spleen and liver, kidnapping it and affecting their biological half-life and biodistribution. A hydrophobic therapeutic agent with a cationic charge may bind to a high amount of plasma proteins, resulting in prompt elimination from the circulation [87,162,165].

Junctions between the cells in the arteries, veins, and capillaries form another IV barrier that keeps our blood flowing but makes it difficult for the drugs to cross, which is a phenomenon known as extravasation [87,162]. Following sustained blood circulation, the therapeutic agent needs extravasation and accumulation in the target tissue or cell. In infectious diseases, the extravasation and accumulation are facilitated because of the EPR phenomenon, characterized by the presence of disorganized leaky vessels with heterogeneous blood flow, allowing the therapeutic agent to cross the endothelial layer and penetrate deep within the interstitial space [166,167]. The therapeutic agent needs to reach the intracellular level required and be endocytosed by cells, following site-specific extravasation. Whereas hydrophobic molecules are capable of diffusing through the lipid bilayer of membrane cells, cationic molecules are more internalized than anionic ones in different cell types. Endocytosis is a common mechanism associated with molecule internalization through membrane invaginations and intracellular vesicles. Depending on the type of membrane protein that recognizes the molecules and physicochemical properties of therapeutic agents, clathrin-mediated, caveolae-mediated and lipid-raft-mediated endocytosis are the pathways [168].

#### 3.2.2. Oral Administration

Oral is the most convenient method of drug administration, as less sterility of the therapeutic agent is required and it is pain-free. Yet, the effectiveness of drugs administrated orally is reduced by both acidic pH and action of enzymatic digestion in the stomach [162,169]. Once they overcome the stomach, molecules can enter the small intestine via duodenum, which, having a large number of digestive enzymes, degrades the molecules allowing its absorption. Herein, the molecules need to traverse several barriers to reach the lumen of the blood vessel [87,162,170], constituted for a large area of villi, where the main cells are enterocytes (or globet cells) and M cells covered by a large layer of mucus. The mucus is produced by globet cells to protect epithelial cells of foreign or physical damage by ingested food. The therapeutic agent needs to reach the intestinal epithelial cell layer to penetrate the mucosal barriers [169,170,171]. Therefore, modulation of the physicochemical properties of the therapeutic agents is necessary to cross the mucus. For example, neutral and anionic molecules overstep the mucus layer to cross later the first line of epithelial cells, which tend to take up more molecules with a positive charge than those of negative charge [87,162,170].

#### 3.2.3. Intranasal Administration

The pulmonary route of administration is relatively complex because the respiratory tract develops mechanical, chemical and immunological barriers to keep inhaled therapeutic agents out of the lungs and remove or inactivate them once they go inside. The therapeutic agent needs access to the large lungs’ epithelial layer but must pass numerous airway bifurcations where it could be deposited [162,172]. To deliver the therapeutic agent into the lungs requires an aerosol size with an aerodynamic diameter [172]. The lungs have a huge presence of mucus, which has a bulk viscosity 105 times greater than water [171]. The lungs’ mucus has a villi layer above, which constantly beats, removing deposited materials from the conduct. If the villi do not remove the drug, actions of proteolytic enzymes and surfactants may hydrolyze it. Additionally, the therapeutic agent can suffer rapid and nonselective clearance by the MPS after deposition in the lungs. [162,172,173]. Thus, the effect of the barriers in the lungs reduces the drug’s bioavailability.

#### 3.2.4. Topical Administration

Administration by the skin is increasingly being used in the application of therapeutic agents due to it being a less invasive route. The skin is the first line of body defense, preventing the entrance of chemical and biological agents. The epidermis is composed mainly of a layer of keratinocytes (95%) and a minority of melanocytes (melanin production cells) and dendritic cells (involved in the immune response), whose composition absorbs lipophilic compounds through the skin. The substances traverse the barrier by transcellular, intercellular, and transappedageal processes (diffusion via the hair follicle’s sebaceous and sweat glands) [174,175]. The transcellular route involves higher resistance because the therapeutic agents must cross different lipophilic and hydrophilic compartments, being the main pathway for polar molecules. The intercellular mechanism is one of the favored pathways to penetrate the barrier because of the epidermal permeability from the physical structure of the intercellular lipids. Small lipophilic molecules across intercellular spaces with diffusion rate are dominated not only by their chemistry nature but molecular weight, and the ability for hydrogen bonding and solubility.

In contrast, large molecules have a physic restriction to penetrate lipid channels. Diffusion through the hair follicle, sebaceous and sweat glands is an alternative pathway to cross SC because their structure provides a niche for mechanical accumulations and storage of substances. Additionally, the easy access to the bloodstream and lymph makes the hair follicle a potential target to administrate therapeutic agents for local or systemic applications, as follicle size is one of the main characteristics influencing the penetration by the hair [174,175].

### 3.3. Therapeutic Challenges

Eukaryotic cells are sub-compartmentalized in membrane-bound structures known as organelles (phagosome, phagolysosome, endosome, endolysosome, nucleus, mitochondria and endoplasmic reticulum), being the target of the intracellular pathogens that survive off proteins and lipids from organelles within the host. Nanosystems to treat intracellular pathogens have the challenge of delivering drugs at the intracellular level in the cytosol or through the specific organelles. The pathogenesis process takes place by invasion, survival, replication, and exiting the host cell. The invasion involves the steps for extracellular to intracellular space transition. After the invasion, some survive by altering antimicrobial mechanisms and others create a compartment, which is impervious to the microbicidal mechanisms of the host cell. For all intracellular pathogens, surviving in a permissive cell leads to replication with an increase in intracellular microbial loading [121], and the subsequent exiting of microbial progeny towards the extracellular space by, for example, spreading from cell to cell or by inducing cell lysis [121,128,176].

Many intracellular pathogens evade the host immune response causing disease by residing and replicating inside the host cells, primarily macrophages. One of the main strategies of microorganisms such as bacteria, protozoa and fungi is the manipulation of the organelle “phagosome” involved in phagocytosis, the main mechanism of defense of macrophages [10,35,124,177]. The phagosome is a degrading organelle developed by the cell to internalize and degrade microbes. Cells first uptake microbes by phagosome, and then a series of synchronic events leads to fusing with lysosomes, forming a structure known as phagolysosome. The phagolysosome contains different enzymes and microbicidal compounds within a low-pH environment that degrade pathogens. Based on the avoiding strategy, intracellular pathogens can be classified in those that avoid interface with phagosome maturation and those that form the phagolysosome as a special compartment for pathogen replication. The first group blocks the formation of phagolysosome but converts the phagosome into a compartment that resembles the organelle providing protection from immune response and promoting the pathogen life cycle. *Mycobateria tuberculosis* [178], *Salmonella* [179], *Coxiella burnetii* [180] and *Histoplasma capsulatum* [119] are typical pathogens that survive inside the phagosome.

The second group uses proteins and lipids from host cells to replicate and some of them can convert the phagosome-lysosome into an organelle similar to Endoplasmic Reticulum (ER) and Golgi apparatus. Whereas *Legionella pneumophila* [179], *Brucella* [180] and *Toxoplasma gondii* [181] can locate within the ER, *Chlamydia* locates into the Golgi apparatus [181,182,183]. Viruses depend on host cell organelles to infect successfully but alter their morphology and functions and cellular processes to complete their life cycle. Such alterations include membrane disruption and fragmentation of the nucleus, relocalization or depletion of host nucleolar proteins, disruption of nucleocytoplasmic trafficking, the formation of single membrane tubules, double-membrane vesicles and vesicles with zippered appearance, as well as the alteration in the post-translational machinery and Golgi bodies [184,185]. Each pathogen has specific strategies to counteract host immune responses by developing a unique intracellular niche reflecting the outcome of and aggressive fight at the host-pathogen interface so that understanding these interaction mechanisms may profoundly impact on the design of improved antimicrobial strategies.

Nanosystems’ surface modification is a good alternative to deliver drugs in a site-specific way. After internalization by endocytic pathways, the main challenges are to reach out to the cytosol by escaping the endolysosome compartments avoiding premature drug release and the acidic pH inside the organelle. One way to achieve disruption of the endolysosome structure and thus, drug release in the cytosol is through pH-response polymers [181,182]. Viruses use the host machinery of the nucleus organelle to replicate the virus’s genetic material. NPs rarely accumulate in this organelle because the nuclear membrane, having a pore size ≈10 nm, is difficult to penetrate, thus preventing the passage of molecules bigger than 40 KDa. Whereas nanosystems smaller than 10 nm can be passively transported, target-ligand functionalized NPs may actively penetrate the nucleus. Nuclear localization signals (NLS), composed of essential amino acid residues, can be recognized by cytoplasmic receptors and subsequently, binds the nuclear membrane [183,184]. The HIV-TAT peptide coating the surface of NPs evades endosomal sequestration, allowing nanosystems’ accumulation in the nucleus. Compounds with high affinity for DNA, such as cationic polymers, increase NPs’ orientation to the nucleus due to their capacity to penetrate the nuclear membrane [184].

The role in the regulation of different functions influencing the intracellular survival of pathogens evading host immunity makes the mitochondria another important targeting organelle. For instance, mitochondria produce ROS that can affect intracellular pathogen survival [183,185]. *Listeria monocytogenes* cause mitochondrial morphological changes such as mitochondrial fission by secreting a pore-forming toxin to overcome ROS [185]. Due to the lipophilicity properties of the membrane and the considerable mitochondrial membrane potential (negative inside), the nanosystems to target mitochondria should have lipophilicity or/and a positive charge. Thus, functionalization with polymers or peptides with a positive charge is the typical strategy to localize the nanosystem within the mitochondria. Besides, nanosystems of 80–100 nm in size are uptaken easily by mitochondria. The ER is another targeting organelle whose critical function that involves protein folding, lipid biosynthesis and control of drug detoxification biochemical pathways [183,186] is affected by pathogens such as viruses and bacteria [183,187,188]. The main strategy for targeting the ER is functionalizing a nanosystem with targeting ligands to the KDEL, a receptor expressed on the ER surface mediated by the protein traffic [189].

## 4. Recent Advances of PNs in the Treatment of Intracellular Infections

Targeted intracellular drug delivery is a major challenge to succeed in nanotherapeutics, where the type of endocytosis process is particularly important to consider for killing the pathogens. In this context, phagocytosis by macrophages plays such an important role in the connection of innate and adaptive immunity by expressing pattern recognition receptors (PRR) on their surface to identify parasites and pathogen-associated molecules, favoring the protection of cells against attack of foreign agents. Macrophages expressing plasmatic membrane receptors mediate important interactions with components of host and with foreign microorganisms to improve host defense, inflammation, homeostasis and modulation of immunity, which made them relevant targets of nanotherapeutics for intracellular infections.

**Figure 5:**
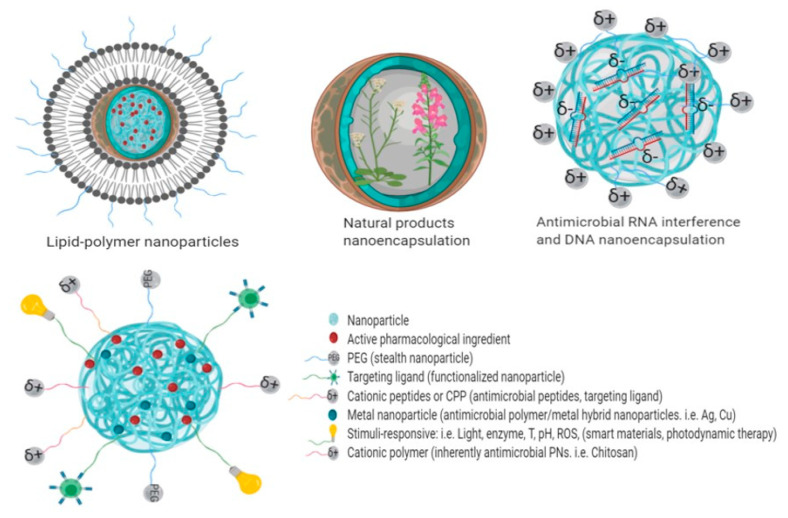
Novel approaches to antimicrobial therapy to attack microorganism resistance.

Receptor-mediated endocytosis is another important strategy widely employed by researchers to design improved nanotherapeutics by using ligands attached to the nanoparticles that can be recognized by the cells for a site-specific targeted therapy [190]. In this sense, there exist a plethora of publications regarding nanoencapsulation of antimicrobials in PNs to treat intracellular infections using diverse approaches to promote endocytosis of the PNs and the killing of pathogens by antimicrobials. These strategies include PNs using polymers as a delivery platform, PNs in which the polymer itself possesses antimicrobial properties and novel approaches such as hybrid platforms, smart materials, ligand-based functionalized PNs, lipid polymer hybrid nanoparticles and antimicrobial photodynamic therapy (Figure 5) [4,46,190,191,192,193,194,195,196,197].

One of the most exciting strategies of functionalized PNs is the use of natural or synthetic polymers with different targeting ligands to provide specificity for organs or tissues, thus improving the uptake of nanoparticles from, for example, the gastrointestinal tract when oral administration is employed, the preferred drug administration pathway among the population. This strategy is relevant, as ligands and certain polymers have shown enhancing oral absorption [198,199]. Moreover, PNs built of stimulus-sensitive polymers and polymers with tuned structural features can be designed with on-demand functions including endosomal scape, high drug loading capacity, prolonged blood circulation and enhanced cellular uptake, among others [58,200].

New efforts to improving antimicrobial therapy include encapsulation of antimicrobial peptides and oligonucleotides in PNs, encapsulation of natural products with antimicrobial properties and nanocrystals of antimicrobials stabilized in a polymeric matrix [109,196,201,202], etc. One typical example of a hybrid strategy involves the encapsulation of metallic nanoparticles with antimicrobial properties within a polymeric platform [203,204]. Most of the strategies use biodegradable and biocompatible polymers and copolymers with PEG and different polyelectrolytes and glyconanoparticles, including CS and its derivatives [4,45,51,60,64,190,201]. Representative examples that show recent advances on PNs to treat intracellular infections are as follows and summarized in Table 3.

Functionalization of PNs with ligands using PCL as polymeric- and building hybrid-platforms with metallic nanoparticles and mesoporous silica has been explored in the drug delivery of antimicrobials and viral therapy [195,203,204,210,215,216]. For instance, Sinko and collaborators prepared nanocarriers made of PCL-b-PEG functionalized with a new ligand 4DV3 used to prove the concept of novel activity against HIV [217]. A bifunctional activity was achieved by targeting the cell surface with the chemokine receptor CXCR4, inhibiting the virus entry and a drug delivery portal facilitating the uptake of nanocarriers by the endocytosis process. Thus, retroviral drugs could be encapsulated in functional nanocarriers to potentiate the therapy against HIV infection.

Curcumin is an active principle with multiple pharmacological activities, but its high hydrophobicity restricts its aqueous solubility and applicability [218,219,220]. Curcumin encapsulated in PLGA nanoparticles has been explored as a new strategy to improve the intracellular delivery of this active principle to improve antibacterial, antiviral, antimycobacterial and antifungal properties [8]. PLGA-curcumin PNs were more efficiently phagocyted by macrophages as compared to free curcumin, demonstrating the potential of this nanoplatform to fight intracellular infections.

Natural products with antimicrobial properties encapsulated in PLGA PNs is a trend in recent studies. For example, Rosin acids (RA) derived from coniferous trees and composed of a mixture of hydrophobic diterpene carboxylic acids, have been used to treat skin infections. Santovito and collaborators recently nanoencapsulated RA within PLGA-PEG PNs and proved its antimicrobial activity against different Gram (+) and Gram (−) pathogens [221]. Nanoencapsulation of RA into PLGA-PEG PNs was a successful strategy to produce aqueous-based formulations of RA with antimicrobial activity.

Hyaluronic acid (HA) is a polysaccharide obtained from natural sources found in microorganism membranes and animal tissues having multiple properties, including inhibition of tumor growth, wound healing and anti-inflammatory properties. HA has been explored extensively in biomedical applications and drug delivery [222,223]. It was used as a ligand to target CD44 antigen, which is expressed in abundant mammalian cells, whose uptake was by endocytosis mediated by the HA receptor. Macrophages express CD44 when starting an inflammatory response as the one generated in infections occurring by pathogens. Therefore, the cellular uptake of HA-functionalized PNs can be accomplished by infected macrophages [224,225]. HA-based nanogels with HA acting as polyanion and PEI as polycation were produced to improve the internalization process in antimicrobial therapy, by exploiting the intrinsic antimicrobial properties of PEI and the multiple functions of HA.

Antimicrobial activity of (HA)-PEI nanogels against Gram (+) and Gram (−) bacteria strains were evaluated in *S. aureus*, *E. coli* and *B. subtilis* showing the strong killing effect of cationic groups from PEI polymer in binding and precipitating some cellular materials such as lipid and nucleic acids [226]. Encapsulation of antimicrobial peptides for tuberculosis treatment using HA as a delivery carrier has demonstrated a significant reduction of the pathogen loading in infected macrophages in vitro with low levels of expression of two pro-inflammatory cytokines (IL-6 and TNF-α), which were associated with reduced intracellular loading of *M. tuberculosis* in infected macrophages [225]. In general, nanogels performance has received considered attention due to their excellent properties of biocompatibility, high drug loading, tunable characteristics to produce stimuli-responsive PNs, controlled and sustained delivery of antimicrobials with exciting perspectives for the management of intracellular infections [60,227,228,229,230].

Another tendency in recent studies about site-specific therapies for infected macrophages inducing an immune response is exploiting the C-type lectin superfamily receptors. They are an essential target to be used in antimicrobial therapy because of their expression on the surface of many intracellular pathogens [190,210,231]. Particularly, the mannose receptor (CD206) has been involved in abundant studies of antimicrobial therapy based on PNs. Besides, the functionalization of PNs with mannose ligands can be accomplished straight forward by the standard carbodiimide coupling chemistry. Studies of nanoencapsulation in mannose-functionalized PCL PNs [210], amphotericin B encapsulated in CS [232] and curcumin [233], itraconazole [10] and amphotericin B [231] encapsulated in PLGA have enhanced the killing of intracellular pathogens with respect to the drug encapsulated into non-functionalized PNs and the drug alone. Research on such new formulations hopefully makes them hit the market shortly. As a sample of this fact, a short description of recently adjudged patents is presented in Table 4. They refer to the fabrication of novel polymers with pendant groups as potential antimicrobial-, photodynamic- and thermosensitive-therapy and antiviral drug delivery systems.

The extensive biological research has contributed to identifying new therapeutic targets. For example, the neonatal Fc receptor (FcRn) is a recently approved patent for improving the oral administration of nanotherapeutics (Patent number: WO2020086871A1) [234,235]. It used PNs functionalized with the Fc fragment of IgG to target neonatal Fc receptor (FcRn) through an FcRn-mediated transcytosis mechanism so the PNs can finally overstep the intestinal epithelium. Valuable information that may be of interest to the reader have also been reviewed elsewhere regarding nanotherapeutics of leishmaniasis [120,236,237], tuberculosis [238,239,240,241,242], viral infections [243,244], HIV [245,246,247,248,249], malaria [118,250,251,252], infectious diseases [4,45,66,253], intracellular delivery [59,60,64,83,254,255], bacterial pathogens [46,192,256,257,258,259] and stimuli-responsive systems [15,58,260,261,262].

## 5. Pharmacokinetics, Biodistribution and Nanotoxicology

Extensive studies in nanomedicine based on the development of new therapies to treat different diseases have been done during the last decade. A growing number of nanosystems have received regulatory approval and the majority were safe, biocompatible and effective. Due to the use of different materials, solvents, shapes, targeting ligands and methods of manufacture, it is imperative to evaluate the toxicity, biodistribution and pharmacokinetics of the nanosystems to reach a safe and effective therapy towards the translational clinic. Even though the PNs have demonstrated significant advantages in the delivery of drugs against intracellular pathogens, they present disadvantages such as the use of organic solvents in their fabrication process, physiological barriers to overstep, poor biocompatibility, immune response activation and cytotoxicity. Understanding the physicochemical characteristics of nanoparticles and how these characteristics affect their interaction with the body is imperative to overcome limitations and in establishing strategies to improve therapy efficiency.

### 5.1. Pharmacokinetics

The pharmacokinetics of PN-encapsulated drugs can be affected by size, shape and materials composition, which impact both the degradation of nanosystems and drug release kinetics. Xu et al. demonstrated that an increased superficial area/volume ratio decreased the drug rate of biodegradation, being lower in rod-shaped (30 days) and tablet (30 days) nanosystems and faster in film (19 days) and microsphere-shaped ones (16 days). The encapsulated drug in films released five times faster than in microspheres [14]. The shape influences the hemodynamic behavior affecting the half time of circulation of the nanosystems by the intravenous administration route, leading to either longer circulation time or no circulation at all. The nanosystems in blood move by fluids, which forces affect their direction and velocity, causing their coalescence against vascular walls, thereby increasing the probability of interaction with macrophages and, therefore, being easily eliminated from blood and body. Nanosystems with non-spherical shapes can persist in the circulation for a longer time than their spherical counterparts [263]. Physicochemical properties of encapsulated drugs also influence drug pharmacokinetics. The molecular weight and hydrophobicity/hydrophilicity properties can modulate drug diffusion across the polymeric matrix. Drugs with high molecular weight have shown longer release delay than drugs with lower molecular weight. For instance, whereas most of the drugs with low molecular weight release in 1–21 days, drugs with high molecular weight release in two bursts between 10–38 days due to a combination of diffusion and degradation processes [264]. The initial release of hydrophobic drugs by burst is much lower because, having low aqueous solubility, they have a strong affinity with the polymer [14]. PEGylation can modify the nanosystem surface, generating a hydrophilic outer shell that decreases the hydration free energy promoting water pore formation and faster drug release. Li et al. demonstrated that a therapeutic agent encapsulated into PLGA-PEG PNs had an initial burst release with a significant amount of therapeutic agent (30.1%) in contrast to PLGA PNs without PEG (20.7%). Both PNs presented a sustained release of the therapeutic agent, but the PLGA-PEG system released it to a higher degree (70%) than the PLGA one (49.8%) [14,265]. Both PNs presented a sustained release of the therapeutic agent, but the PLGA-PEG system released it to a higher degree (70%) than the PLGA one (49.8%) [14,265].

Physiological pH can modulate the polymer degradation rate, affecting the drug release rate, i.e., strong acidic and basic environments accelerate PLGA degradation, thus increasing the drug release rate [266]. Additionally, the sterilization process can alter the PNs’ internal structure and, therefore, the drug release and γ-irradiation may also lead to a random cleavage of the polymeric chains, accelerating its degradation and drug release [267].

### 5.2. Biodistribution

One crucial issue to improve PNs’ biodistribution is the capacity to cross physiologic barriers, which can be modulated by the physiochemical characteristics of the PNs. The main physiological barriers that the PNs need to overstep are the cells belonging to RES and MPS, especially the macrophages of RES, which promote a high biodistribution of PNs in the liver or even cause a rapid elimination of them from the body.

**Figure 6:**
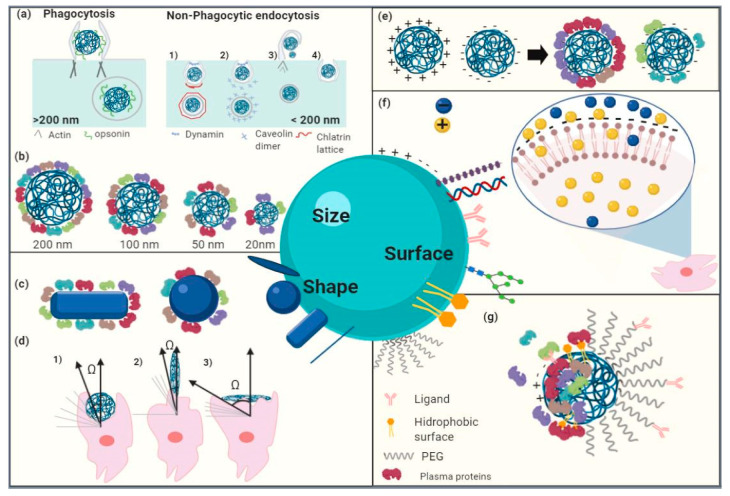
Physiochemical properties of PNs influenced by physiological conditions (and vice versa). (**a**) Size effect on endocytosis pathway. Large particles are uptaken by phagocytosis and smaller particles by non-phagocytic endocytosis, i.e., chlatrin-mediated endocytosis (a1), caveolar-mediated endocytosis (a2), macropinocytosis (a3), and chlatrin- and caveolin-independent endocytosis (a4). (**b**,**c**,**e**), Size and shape effect, superficial area increases adhesion of plasma proteins. (**d**) PNs shape module the macrophages’ phagocytosis, which lead to internalization or simply spread on particles: when Ω is less than or equal to 45°, particles are internalized successfully (d1, d2), when Ω is greater than 45°, cells spread on the particle and internalization is inhibited (d3). (**f**) Surface charge impact PNs’ cellular uptake, cells uptake easier with cationic particles that interact with anionic charges of the cell membrane. (**g**) Hydrophilic steric barrier and neutral superficial charge of PEGylated PNs decreases plasma protein adhesion.

The recognition of PNs by these types of cells can be modulated by size, shape, surface charge and hydrophobicity/hydrophilicity of PNs because such characteristics regulate the interaction with physiological proteins and the protein corona formation. The type of proteins from the protein corona determine the PNs biodistribution or their fast elimination from the body. PNs size, shape, and superficial charges affect the number of plasma proteins that adhere to the nanosystems surface and, therefore, the cells uptaking. PNs with a positive charge, big size and nanorods shape adhere more proteins than smaller nanosystems and they are easier uptaken by cells (Figure 6b,c,e) [263,268,269]. The nanosystems size affects macrophages’ endocytosis pathway. Whereas PNs higher than 200 nm are uptaken via phagocytosis (Figure 6a), PNs smaller than 200 nm are uptaken via non-phagocytic endocytosis pathways [268]. Furthermore, the shape can affect nanosystem phagocytosis by macrophages, i.e., the nanoparticle-cell contact point determines whether macrophages initiate internalization or are simply spread on nanosystems in which the Ω angle is a crucial criterion (Figure 6d). The spherical shape is then an ideal shape to induce phagocytosis of the nanosystem since it has Ω angle of less than 45° [268,270].

Similarly, the chemical polarity of the nanosystem promotes cellular uptake, which is also involved in the protein and lipid adhesion. For instance, hydrophobic nanosystems adhere a higher amount of plasma proteins and have a high affinity for the hydrophobic lipids of the membrane cell, increasing the probability of cellular uptake (Figure 6g). PEGylation has been used not only to neutralize the nanosystem charge but to provide hydrophilic properties, steric hindrance stabilization, reduce the plasma proteins’ adherence and unspecific phagocytosis, and increase the specificity of targeting ligands (Figure 6g).

Size plays an essential role in the biodistribution of PNs. PNs of smaller sizes have the tendency to aggregation, decreasing their capacity of biodistribution. PEGylation reduces the PNs’ aggregation, avoiding their accumulation in non-targeted organs. Biodistribution of nanosystems administered by the intranasal route is highly particle size-dependent. In general, inhaled PNs with aerodynamic diameters between 0.5 and 5 µm are deposited in the central and distal tracts, while those with a size >5 µm are trapped in the upper airways (i.e., mouth, trachea and bronchi) and those with size <0.5 µm are mostly exhaled. Besides, modification of the PNs’ surface may help to modulate the deposition pattern in the lungs [174]. Ungaro et al. demonstrated that a PVA modified alginate/PLGA nanosystem reached the deep area of lungs, while a CS modified alginate/PLGA nanosystem was found in the upper airways and lining of lung epithelial surfaces [271]. Nanosystems administrated by the oral route are eliminated by the efflux pump of P-glycoproteins from intestinal cells, preventing the nanosystems from reaching the blood and distributing to other organs.

PNs can be modified with bioadhesive materials such as PVA, PEG, and vitamin E-TPGS, among others, to improve the adhesion interactions and enhance the internalization into the intestinal cells as well as the ability to escape from efflux pump proteins [272,273]. A high percentage of PLGA nanosystems distribute in the liver, followed by kidney, brain and heart. The PLGA nanosystems can cross cellular barriers such as the blood-brain barrier (BBB) and reach hard-to-target tissues. Whereas after seven days of treatment, the nanosystem remained detectable in the brain, heart, kidney, liver, lungs and spleen, a low quantity was in the spleen and a high extend was localized in the liver (40.04%), kidney (25.97%) and brain (12.86%) [274].

The main option to regulate the biodistribution through a target cell or tissue is by targeting ligands. Resident macrophages are present in each organ from the body, having different characteristics and functions. They adapt to the tissue location, which impacts on their biological function. The expression of different receptors characterizes the macrophage population of each organ. For example, lung macrophages in mice (alveolar macrophages) have high expression of mannose and siglec-F receptor and low expression of F4/80 receptor as compared to peritoneal macrophages that present an intermediate expression of F4/80, low expression of mannose and no expression of siglec-F receptor [275]. Mannose-functionalized PNs have demonstrated to control Leishmaniasis infection by increasing the distribution of the functional PNs in the affected organs such as liver and spleen and decreasing the amount in peripheral blood as compared to PNs without mannose [231,233]. Cell-penetrating peptides (CPPs) as targeting ligands facilitate cellular intake. CPPs are characterized by being cationic, relatively short (10–40 amino acids) and facilitate interaction with phospholipidic membranes whose activity can vary depending on their primary sequence [276,277,278]. Natural or synthetic CPPs such as arginine enhance the cellular uptake of proteins, nucleic acids, drugs, and nanoparticles. Yongjian Liu et al. developed a nanohydrogel functionalized with arginine (Arg 9) CPP to assess lung retention and cellular uptake after intratracheal administration in vivo. Arg 9 peptide promoted the restricted PNs’ biodistribution in the lung, having prolonged lung retention. PNs’ uptake was predominant in alveolar macrophages and lesser in lung epithelial cells. Overall, the type of targeting ligand plays an important role in the intracellular biodistribution of PNs, depending on the ligand nature, the nanocarrier can remain inside the endosome or escape from it to reach other organelles, the cytoplasm or the nucleus [278].

The specificity of targeting ligands can be affected by its density and orientation onto the nanoparticle surface, thus, impacting its biodistribution. Functionalization methods may modulate the density and orientation of molecules or by masking the recognition region from cell bioreceptors [279]. For instance, antibodies can be immobilized on PNs by reversible or irreversible methods. Physical adsorption is the most widely used reversible method, where the union occurs through ionic, hydrophobic or Van der Waals weak interactions. Although this method can produce well-oriented molecules on top of PNs, it may have poor reproducibility and low stability at different pH conditions. There are several options for irreversible functionalization by covalently linking, depending on the reactive groups onto the PNs’ surface. Among them, the most extended is the carbodiimide amine-carboxyl group coupling reaction [279]. It is widely used due to its high stability, but it can present aggregation, polymerization and random orientation of molecules, affecting their accessibility and the binding to recognition sites from cells. Tonigold et al. showed a pre-adsorption process to attach targeting antibodies to the nanosystem surface in the proper orientation, where the nanosystem remained functional and was not entirely covered by protein corona. In contrast, immobilization of antibodies by the carbodiimide method lacks a correct orientation and the functional PNs are more affected by the shielding of the protein corona [279].

### 5.3. Nanotoxicology

Before advancing towards in vivo applications, it is necessary to test possible toxic effects of PNs on human health related to their physicochemical properties. One of the main purposes is to study the nanosystem immunotoxicity. Reports have associated the high level of proinflammatory cytokines upon nanosystem treatment with low therapeutic efficacy, side effects and toxicity. The level of toxicity of PNs depends on the size and administration dosage. Negative charged PNs can activate the immune response, whose activation is related to pseudoallergy; and increased hemolysis. Neutralizing the charge by surface modification of the nanosystem with other polymers or materials prevents binding of protein corona to the nanosystem and then activation of the immune response. Tirtatmadja et al. observed that a variation of the protein corona composition at the nanosystem affected the production of IL-8 and TNF-α inflammatory cytokines [280]. Benita et al. demonstrated that cationic group-coated PNs activated the immune response (inflammation) more than anionic group-coated PNs. PNs with cationic groups on surface presented side effects in lungs and transient systemic toxicity because they are more prone to being captured and bound to sputum components [173]. Particularly, dendrimers presented cytotoxic and hemolytic properties related to the core chemistry, but mainly to the surface end-group [281]. Most of the dendrimers have a strong cationic charge, presenting a strong interaction with the negatively charged cell membranes, causing cell destabilization with leakage of cytoplasmic proteins and subsequent lysis. Surface modification of dendrimers is a promising alternative to improve their safety, such as PEGylation or shielding of their cationic charge by acetylation and hydroxylation [282,283]. Activation of the immune cells depends on the superficial functional groups of PNs. Fuchs et al. found that nanoparticles with amino groups decreased the phagocytosis in macrophages and carboxyl groups increased the production of molecules such as TGF-β1 and ATP level and both nanoparticles impaired expression of some receptor (CD163 and CD200R) in macrophages [284]. Nanoparticles with amino groups also activated a complex of intracellular proteins, activating a subsequent release of pro-inflammatory molecules such as IL-1β [285].

Hydrophobicity properties and size influences PNs’ toxicity. PNs with a high hydrophobicity induced acute respiratory toxicity upon single-dose administration [286]. Smaller PNs increased the dispersion to the nucleus steadily, causing intrinsic toxicity at both the cellular and systemic level [287]. Modulation of size is crucial, depending on the administration route. For instance, submicron particles are necessary to deliver them by the intravenous route and avoid pulmonary embolism associated with nanostructure sizes larger than 5 µm [173]. Impurities from the PNs’ formulation, such as residual solvents, sub-products or endotoxin contamination, may activate the immune system, especially the ROS production, generating an inflammatory response [288].

Despite the multiple reports highlighting the properties of PEGylation to reduce an immunogenic response, many publications have demonstrated that PEGylation may cause immunogenic effects in some individuals, inducing the production of antibodies, which enhance blood clearance, reduce the efficacy of nanotherapeutics and increase side effects [289,290,291]. Studies have shown the absence of cytotoxic effects of PLGA PNs when tested in vitro and in vivo. An in vivo model demonstrated that after seven days of treatment, a high concentration of PLGA PNs caused no morphological pathology in the tissues and histopathology showed neither lesions nor inflammation patterns [274].

## 6. Future Outlooks

Attempts to fight intracellular infectious agents have given us important lessons, bringing out how urgently needs to be the efforts to develop alternative antimicrobial therapies that manage to increase antimicrobial efficacy and decrease microbial resistance as compared to conventional antimicrobial treatments. In this context, PNs have emerged as a nanoplatform having broad prospects for the development of highly promising site-directed antimicrobial therapies for the management of intracellular infections whose products on the market expect to be established shortly, as judged by the number of research publications and patents currently granted in the field.

Recent advances in nanoparticulate formulations, including hybrid strategies, nanoencapsulation of natural products, targeting ligand-based formulations and smart materials, have shown to allow tuning the bio-physicochemical properties of PNs for enhanced antimicrobial efficacy. However, there are still many challenges to face to improve PN-based antimicrobial technology towards scaling-up at the industrial level and reach the market. For example, PNs captured by the immune system hinders the site-specific drug delivery facilitated by highly selective targeting ligands leading to ineffective internalization. Nanosystems toxicity and nonspecific biodistribution limit in vivo practical applicability. Furthermore, proper standardization is not trivial both in vitro and in vivo, which limit systematic comparative studies. Therefore, it is necessary to continue researching to advance the limited understanding of the fundamental processes of PNs in and out of the human body, given the multiple interactions of antimicrobial nanotherapeutics among them, with the protein corona and with the vast diversity of organs, tissues and cells on their journey from administration to therapeutic targets.

A higher number of standardized production methods, validated studies of toxicity, bioequivalence, clinical studies, and the establishment of reference materials may impact on gaining a better knowledge of the antimicrobial nanotherapeutic systems and their associated pro and contra, in the way to creating products that ensure quality, safety and efficiency. The final balance of this process defines the scope of PN-encapsulated drugs, to quickly reach high acceptance in the market, thereby offering added value as compared to conventional antimicrobial therapies.

## Figures and Tables

**Table 1:** Therapeutic principles to treat intracellular infections.

Classification	Example	Target	Action Mechanism	Secondary Effects	Ref.
Antibiotics	β-Lactams	Bacterial cell wall	Inhibiting bacterial cell wall synthesis.	Allergy, diarrhea/colitis, pruritic rash, abnormal coagulation, abnormal liver function. Drug-drug interaction with bacteriostatic antibiotics displaying an antagonized effect.	[136]
	Polymixins	Bacterial cell wall	Positively charged polymyxins bind to molecules of the bacterial cell wall with negative charges, affecting the osmotic balance.	Nephotoxicity, paresthesias, apnea, nausea, vomiting, dizziness, myopathy and neuropathy.	[132,137]
	Quinolones and Fluoroquinolones	Enzymes	Inhibits DNA gyrase and topoisomerases enzymes.	Nausea, vomiting, dyspepsia, heartburn, abdominal pain, inflammation in tendons, musculus and joints, depression, affects memory and sleeping.	[136,138]
	Sulphonamides and trimetroprim	Metabolic pathways	Interferes with folic acid synthesis by preventing the addition of para-aminobenzoic acid (PABA) into the folic acid molecule.	Rashes, erythema modosum, dermatitis, photosensitivity, acute hemolytic anemia, agranulocytosis, aplastic anemia, liver injury, hepatic granuloma. Drug-drug interaction with bone marrow depressants increases the leukopenic and/or thrombocytopenic effects.	[132,136]
Antivirals	Acyclovir	DNA polymerases	Chain terminator.	Pain, swelling, abdominal or stomach pain, loss appetite, nausea or vomiting and reversible nephrotoxicity.	[139]
	Enfuvirtide	Blocks virus entry to the host cell	Inhibits glycoproteins that mediate the membrane fusion of virus.	Rash, fever, nausea, vomiting, chills, rigors, hypotension and elevation of serum liver transaminase level.	[140,141]
	Oseltamivir	Blocks release virions from infected cells	Inhibits glycoproteins that mediate the release of virion.	Nausea, vomiting, bronchitis, insomnia, vertigo, abdominal pain, epistaxis, optic disorder and conjunctivitis.	[142,143,144]
	Interferons (IFN)	Immune response	Inhibits virus replication, suppression of cell proliferation, enhancement of macrophages’ phagocytic activity.	Fever, fatigue, bone marrow suppression, influenza-like symptoms, depression, development of autoimmune illnesses, cardiovascular disorders, thyroid disorders, dyspnea and pneumonitis.	[139,145,146]
	Efavirenz	Inhibition of reverse transcriptase	Binds to a region that is distinct from the dNTP-binding site on the viral RT protein.	Abnormal dreams, abnormal thinking, agitation, amnesia, confusion, depersonalization, dizziness, euphoria, hallucinations, insomnia, somnolence and serum aminotransferase elevation.	[147,148]
Antifungals	Amphotericin B	Fungi cell wall	Binds to ergosterol in the fungal cell membrane, disrupting cell permeability.	Nausea, fever, pulmonary toxicity, abdominal pain or leg pain, nephrotoxicity, hemolysis and liver damage.	[36,149]
	Azole agents	Fungi cell wall	Inhibits cytochrome P-450 dependent enzymes needed to synthesize ergosterol of the fungal cell membrane.	Nausea, diarrhea, hypertension, hypokalemia, edema, liver injury and hepatotoxicity; drug-drug interaction, inhibits the cytochrome P450 (CPY450) enzymes in the liver and interacts with P-glycoproteins (P-gp) in the cell membrane involved in the absorption and distribution of drugs, affecting the therapeutic response and the interaction with other drugs.	[39,150,151]
	Echinocandins	Fungi cell wall	Inhibits the production of (1r3)-β-d-glucan, an essential component in the fungal cell wall.	Fever, nausea, vomiting, headache, pain, rash, anemia, abdominal pain, serum aminotransferase elevation.	[150,152,153]
Antiparasitics	Chloroquine	Inhibition of metabolic pathways	Inhibits the parasitic heme detoxification and nucleic acid biosynthesis.	Apnea, nausea, vomiting, cramps, diarrhea, hepatotoxicity, cardiotoxicity.	[154,155,156]
	Pentavalent antimonial	Parasitic cell wall	Inhibits glycolysis and b-oxidation of fatty acids of parasite.	Pancreatitis, pancytopenia, reversible peripheral neuropathy, elevation in serum aminotransferases, pain, stiff joints, gastrointestinal problems, hepatic-, renal-insufficiency (nephrotoxicity), cardiotoxicity, accumulation inside the tissues of liver and spleen.	[157]
	Pentamidine	Interferes with nuclear metabolism	Inhibits synthesis of DNA, RNA, phospholipids, and proteins.	Skin rash, nausea and vomiting, anxiety, headache, hypoglycemia, hypertension, myalgia, and headache.	[130,131,132,133,134,135,136,137,138,139,140,141,142,143,144,145,146,147,148,149,150,151,152,153,154,155,156,157]

**Table 2:** Some examples of antimicrobials resistance.

Antimicrobial (Disease)	First-Line of Treatment	Second-Line of Treatment	Resistance Cause or Mechanism	Resistance Characteristic and Consequence	Ref.
Antibiotic (Tuberculosis)	Rifampin, Isoniazid, Pyrazinamide and Ethambutol	Fluoroquinolons, aminoglycosides, para-aminosalicylic acid, and cycloserine	Poor solubility, low plasma levels, low permeability and are rapidly metabolized by the liver, thus requiring multiple and high doses.	Multi-resistant strains appear by interruption of the treatment schedule; therapy becomes more toxic and less effective, drugs are more expensive and scarcer. Neurotoxicity and hepatotoxicity.	[30,37,158,159]
Antiviral (VIH)	Disoproxil fumarate, Lamivudine or Emtricitabin, Efavirenz	A boosted protease inhibitor (bPI) plus two nucleoside analogues (NRTIs)	Poor treatment compliance, variable drug pharmacokinetics, pharmacokinetic interactions and pharmacodynamics, thereof, low penetration into certain body compartments, leads to subtherapeutic levels and, therefore, to the selection of resistant viruses.	Treatment failure and further spread of drug-resistant HIV. It can compromise the effectiveness of the limited therapeutic options and further reduce HIV incidence, mortality and morbidity.	[141,142,160]
Antifungal	Fluconazole	Itraconazole	Drugs have poor solubility, low plasma levels, low permeability. The use of inadequate dosages, when treatment courses are not long enough. The use of fungicides in agriculture contributes to resistance.	Therapy becomes more toxic and less effective. Hepatotoxicity.	[150,152,161]
Antiparasitic (Leishmaniasis)	Pentavalent antimony	Pentamidine	Exposure of low concentration of arsenic leads to the emergence of parasite resistance.	Adjusting doses, frequencies and administration time to maintain its efficacy but with an increase in the severity of the side effects.	[120,157]

**Table 3:** Some recent studies that use PNs to treat intracellular infections.

Function	Formulation	Drug/Active Principle	Targeted Microorganism or Cells	Method of Fabrication	Outcome	Ref.
*Conventional PNs*						
Antibacterial	PLGA	Gentamicin	*Klebsiella pneumoniae*	Water-oil-water (w/o/w) double emulsion method	Reducing bacterial viability without concomitant stimulation of inflammatory or pyroptotic pathways in the treated cells.	[205]
	N-trimethyl chitosan	Recombinant urease	*Brucella melitensis*, *Brucella abortus*	Ionic complexation	Intraperitoneal vaccination with TMC/urease nanoparticles provides more protection and immune response against brucellosis as compared to TMC/urease NPs’ oral administration.	[206]
	β-cyclodextrin	Ethionamide and BDM43266	-	Cross-linking	Co-encapsulation of ethionamide and BDM43266 antitubercular drugs was achieved into β-CyD PNs with the possibility of pulmonary administration.	[207]
Antiprotozoal	TPGS, Tetronics T904 and T1107	Miltefosine	*Leishmania major*	Self-assembly	Nanoencapsulation of miltefosine in polymeric micelles of TPGS, T904 and T1107 enhanced antileishmanial activity as compared to miltefosine solutions. T904 formulation increased activity against intracellular amastigotes of *L. major*.	[208]
Antiretroviral	m-PEG-PLL	Efavirenz and Elvitegravir	TZM-bl cell line infected with HIV	Self-assembly	Enhancing of combined therapy against HIV infection was achieved by encapsulation of antiretrovirals into hydrophobic core graft-copolymer nanoparticles made of m-PEG-PLL with a hydrophobic core of fatty acids with low cytotoxicity and improved biodistribution.	[209]
*Novel approaches on PNs*						
Macrophage targeting	PCL-PEG/MRTL (Bivalent mannose receptor targeting ligand)	-	Rat peritoneal macrophages	Flash nanoprecipitation	Macrophages of M2-type can be targeted using PNs coupled with a new bivalent mannose targeting ligand via mannose receptor, enhancing specificity and cellular uptake of PNs.	[210]
Antifungal	PLGA/DMSA	Itraconazole	*Paracoccidioides brasiliensis*	Emulsification-evaporation method	Nanoencapsulation of ITZ in functionalized PLGA/DMSA nanoparticles improved biodistribution and antifungal efficacy against *Paracoccidioides brasiliensis* evaluated in vivo in infected BALB/c mice as compared to free ITZ, lowering the number of administrations and side effects.	[39]
Antiprotozoal	PLGA/Mannose	Itraconazole	*Leishmania (L) infantum*	Nanoprecipitation	PLGA NPs with mannose receptor increased efficacy against *Leishmania (L.) infantum* amastigotes.	[10]
	Grafted Chitosan/tri-mannose ligand	-	Human macrophages with *M. tuberculosis*	Nanoemulsion and Ionic gelation	CS nanocapsules grafted with tri-mannose ligands modulated cell metabolism of cells infected with *M. tuberculosis*, offering the possibility to reprogram immune cells improving drug therapy.	[79]
	PEG-PPS functionalized with acid-sensitive fluorophores (ASF)	Antigen mycolic acid	*M. tuberculosis*	Self-assembly	Pulmonary delivery of mycolic acid-lipid antigen encapsulated in polymeric micelles enhanced immune response of T cells in mice model hCD1Tg (humanized CD1 transgenic mice) improving immunization therapy against *M. tuberculosis*.	[211]
	N-trimethyl chitosan/poly(trimethylene carbonate) composite	Vancomycin	*S. aureus*	Ionotropic gelation	VCM/TMC NP-PTMC inhibits bacteria and promotes bone repair in vivo.	[212]
Antibacterial	PLGA/PLGA-PEG/Zwitterionicchitosan/Eudragit E100	Vancomycin	Methicillin-resistant *S. aureus*	Water-oil-water (w1/o/w2) double emulsion method	Better antimicrobial activity than free vancomycin against intracellular MRSA and other intracellular pathogens.	[213]
	PLGA/membrane of extracellular vesicle	Vancomycin and rifampicin	*S. aureus*	membrane-coating technique	Membrane-coating had an active targeting capacity and the formulation improved efficacy to treat *S. aureus.*	[194]
	PVA/NaAlg	Amoxicillin	*S. aureus*	Coacervation	Antimicrobial activity is comparable to pure Amoxicillin. pH-controlled release of Amoxicillin.	[214]
	Glycol chitosan-LPNs (PLGA/DDA/TDB)	Antigen CTH522	*C. trachomatis*	Single emulsion evaporation method O/W	Glycol CS-lipid polymer hybrid nanoparticles (LPNs) made of PLGA/DDA/TDB used as adjuvant of antigen CTH522 against *C. trachomatis* in mice model via nasal administration induced IgG/IgA responses increased in lungs and genital tract as compared to adjuvant DDA/TDB liposomal.	[193]
	PCL/SBA-15	Thymol	*S. aureus*	Electrospinning	Antimicrobial activity is better than free thymol against *S. aureus* using rod-shaped particles.	[215]
	PCL/MCM-41 surface functionalized	Gentamicin	-	Electrospinning	Controlled release of Gentamicin and biocompatible material.	[195]

**Table 4:** Some recent patents adjudged in 2020 about PNs to treat intracellular infections.

Patent Name	Polymers	Therapeutic Use	Patent Number
Antiviral prodrugs and nanoformulations thereof	The prodrug, amphiphilic block copolymers, P407	Retroviral, viral, HIV infections	WO2020086555A1
Nanoparticle encapsulation to target G protein-coupled receptors in endosomes	DIPMA-DEGMA-b-PEGMA-DMAEMA; BMA-b-PEGMA-DMAEMA	Delivery of therapeutic principles	WO2020084471A1
Functionalized nanoparticle formulations for oral drug delivery	Different types including polyalkenes, polyesters, functionalized with FcRn binding partner	Various uses including encapsulation of antibacterial and anticancer agents	WO2020086871A1
Polymeric nanoparticles in a thermosensitive gel for coital-independent vaginal prophylaxis of HIV	PLGA, PCL, Pluronic F127, Pluronic F68	Prevention of HIV infection	US2015/O190398A1
Organosilanes for the treatment of infections	Organosilicon quaternary ammonium compounds	Bacterial, fungal, viral infections	WO2020082026A1
Novel nanoparticles of antiretroviral drugs, their preparation and their use for the treatment of viral infections	Chitosan	HIV, viral infections	EP3653201A1
Methods and composition for treating microbial infections	PLGA, PCL, mPEG-PLGA, PVA, PEO, PVP and combinations thereof	HIV, viral infections	WO2020097062A1
Synthetic innate immune receptor ligands and uses thereof	PLGA	Vaccine therapy	WO2020082162A1
Polymer-particle light-cleavable carrier systems for photodynamic therapy	Polycarbonates, polyesters, various types	Infection diseases	WO2020064701A1
Small polymeric carriers for delivery of agents	Hydrophobic polymeric backbone with a plurality of pendant groups	Antiviral infections and delivery of active agents	WO2020077170A1
Antimicrobial compositions and methods	Various type of block copolymers including polyethylene oxide-polyglutamic acid-phenylalanine	Antimicrobial therapy	WO2020056114A1
